# Combination of paclitaxel, bevacizumab and MEK162 in second line treatment in platinum-relapsing patient derived ovarian cancer xenografts

**DOI:** 10.1186/s12943-017-0662-3

**Published:** 2017-05-30

**Authors:** Francesca Ricci, Federica Guffanti, Giovanna Damia, Massimo Broggini

**Affiliations:** Laboratory of Molecular Pharmacology, IRCCS - Istituto di Ricerche Farmacologiche “Mario Negri”, via Giuseppe La Masa 19, 20156 Milan, Italy

**Keywords:** Ovarian cancer, Second line, Patients derived xenografts, Cisplatin, Drug resistance, Combination chemotherapy

## Abstract

Advanced ovarian cancer is very responsive to first line platinum therapy, however almost invariably it relapses with a resistant disease. We have reported that patient derived ovarian xenografts (PDXs), independently from the degree of the initial response to cisplatin (DDP), show a significantly lower response to a second DDP cycle. We here report the effect of new combination regimens containing a MEK inhibitor (MEK), bevacizumab (BEV) and paclitaxel (PTX) as second line therapy in platinum-relapsing PDXs.

We selected three DDP-relapsing PDX models based on the presence of activation of the RAS/RAF/MEK/ERK axis, mutated p53, lack of PTEN expression and activation of the PI3K pathway. In all the selected xenograft models, the antitumor efficacy of the doublets can be summarized as PTX/BEV > BEV/MEK > PTX/MEK and the antitumor activity of the triple combination was higher than any double combination. All the different combinations were well tolerated. The present data corroborate the activity of bevacizumab in combination with chemotherapy for the treatment of relapsing ovarian tumors and suggest that the addition of another targeted agents (MEK inhibitor) can further increase the antitumor activity without any increase in toxicity. PDX models represent a useful model to test second line therapy after failure of DDP first line.

**Electronic supplementary material:**

The online version of this article (doi:10.1186/s12943-017-0662-3) contains supplementary material, which is available to authorized users.

## Background

Epithelial ovarian cancer (EOC) is one of the most deadly tumor of woman, causing roughly 100,000 deaths/year in western countries [1]. Advanced ovarian cancer, which represents more than 70% of all EOC, is well responsive to first line therapy (mainly consisting of platinum-based drugs after cytoreductive surgery), however almost invariably relapses with a tumor no more responsive to platinum-based therapies. Patients are then treated in second line with different combination treatments, including topotecan, trabectedin, pegylated liposomal doxorubicin and recently bevacizumab [2–7], although the response and overall survival (OS) after relapse are far from being satisfactory [8]. Antiangiogenic therapies, and in particular bevacizumab has been recently approved in second line treatment in ovarian cancer patients after relapse to platinum-based therapies [9].

There is an urgent need to find new therapeutic approaches for ovarian tumors relapsing after a platinum based therapy. In the last years, patient derived xenografts (PDXs) of ovarian cancers, based on the transfer of primary tumors directly from patients to immune-deficient mice, have been generated and characterized [10–13]. These PDXs not only recapitulate the tumor of origin in terms of clinico-pathological characteristics and genetic alterations, but also show resistance patterns similar to that observed in the clinic. These models represent translational models to possibly study precision medicine approaches to increase the prognosis of ovarian patients and in particular we think will be instrumental to better understand and overcome platinum resistance. We have recently reported that in several models, independently from the degree of the initial response to cisplatin (DDP), re-growing tumors show a significantly lower response to DDP-based treatment [14]. These in vivo settings represent good models to study new therapies and combinations in these challenging condition, i.e. re-growing tumors after an initial platinum response [10–14].

We here reported the effect of new combination regimens consisting of doublets or triplets, containing inhibitor of MEK (MEK162), placlitaxel and/or bevacizumab in platinum-relapsing ovarian PDXs.

### Findings

All the experiments were performed in female NCr-nu/nu mice (6 weeks old) obtained from ENVIGO RMS srl (Correzzana, Italy) maintained under specific pathogen free conditions, housed in isolated vented cages, and handled using aseptic procedures. The IRCCS-Istituto di Ricerche Farmacologiche Mario Negri adheres to the principles set out in the following laws, regulations, and policies governing the care and use of laboratory animals: Italian Governing Law (D. lg 26/2014; Authorization n.19/2008-A issued March 6, 2008 by Ministry of Health); Mario Negri Institutional Regulations and Policies providing internal authorization for persons conducting animal experiments (Quality Management System Certificate- UNI EN ISO 9001:2008 – Reg, No.6121); the NIH Guide for the Care and Use of Laboratory Animals (2011 edition) and EU directives and guidelines (EEC Council Direcrive 2010/63/UE). The Statement of Compliance (Assurance) with the Public Health Service (PHS) Policy on Human Care and Use of Laboratory Animals was recently reviewed (9/9/2014) and will expire on September 30, 2019 (Animal Welfare Assurance #A5023-01).

The three ovarian PDX models used for these experiments (MNHOC 124, MNHOC 218 and MNHOC 239) have been described in details [10, 14]. The tumors, re-growing after a DDP treatment, were selected on the basis of their growth, expression of markers and response to platinum (Table 1 and Additional file 1: Figure S1). Specifically, tumors were implanted subcutaneously in nude mice and when the tumors reached approximately 150 mm^3^ were treated with DDP 5 mg/Kg iv q7dx3. This regimen induced a roughly 90% reduction in tumor growth in all the three selected models. However, after a variable period, the tumors re-started to grow and were much less responsive to a subsequent cycle of DDP (Additional file 1: Figure S1). These re-growing, platinum-relapsing tumors were excised and re-implanted in additional mice to test, in a second line-like setting, the activity of the combinations under investigation. As for the “first line” setting, when the relapsing tumors reached approximately 120-150 mm^3^, they were randomized to receive vehicle, paclitaxel (PTX, 20 mg/Kg iv q7dx3) plus bevacizumab (BEV, 5mg/Kg ip q7dx3), PTX plus MEK162 (MEK, 3.5-10 mg/Kg po bid daily for 14 days), BEV plus MEK or PTX plus BEV plus MEK. Tumor growth was measured with a Vernier caliper every two-three days, and tumor weights (mg = mm3) were calculated using the formula: (length [mm]*width [mm]^2^)/2. The efficacy of the treatment was expressed as best tumor growth inhibition [%T/C = (tumor weight mean of treated tumors/tumor weight mean of control tumors)*100]. Toxicity was monitored recording animal weights and physical examination every day for all the duration of the experiment.

**Table 1:** Characteristics of the three PDXs used

PDX ID	DIAGNOSIS	*TP53* status	IHC		
			PTEN	NF1	p-ERK
MNHOC124	Serous/endometroid	mut	-	++	+++
MNHOC218	Endometroid	mut	-	+	++
MNHOC239	Serous	mut	-	++	++

*IHC* Immunoistochemistry. Protein expression was cytoplasmic with no nuclear or membrane-associated staining. The intensity of the staining has been ranked according to the following scale: - negative; +/- slight/doubtful; + slight; ++ moderate; +++ marked

Mut: mutated

The immunohistochemical evaluation of NF1, PTEN, ERK and pERK was performed in four μm sections from each tumor xenograft, which were incubated with the following primary antibodies: rabbit polyclonal antibody to NF1 (Abcam, ab30325), rabbit monoclonal antibody to PTEN (Cell Signaling, 138G6), rabbit monoclonal antibody to ERK1 (Epitomics, 1172-1), rabbit monoclonal antibody to phospho-ERK1/2 (Cell Signaling, 4370) and incubated with biotinylated secondary antibody (goat anti- rabbit). Sections were labeled by the avidin-biotin-peroxidase (ABC) procedure with a commercial immunoperoxidase kit. The immunoreaction was visualized with 3,3’-diaminobenzidine (DAB) substrate and sections were counterstained with Mayer’s hematoxylin.

All the three models have an activation of the RAS/RAF/MEK/ERK axis with high expression of NF1 and high phosphorylation of ERK, display a mutated p53 and lack PTEN expression, leading to activation of the PI3K pathway.

**Fig. 1:**
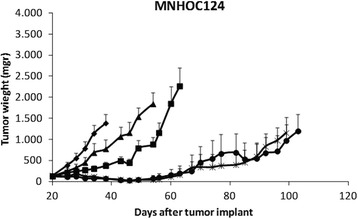
Antitumor activity of the combination of PTX, bevacizumab and MEK162 in MNHOC124 PDX model. Xenograft MNHOC124 was transplanted subcutaneously and when tumor masses reached 100-150mg, mice were randomized to receive vehicle (CTR,-♦-), or bevacizumab and MEK162 (BEV/MEK,-■-), paclitaxel and MEK162 (PTX/MEK,-▲-), paclitaxel and bevacizumab (PTX/BEV, -●-), or paclitaxel and bevacizumab and MEK162 (PTX/BEV/MEK,-x-). Data are the mean ± SE of tumor masses, as described in Materials and Methods; each group consisted of 8-10 mice

Figure 1 shows the tumor growth of all the different combinations and control (no-treated) group in MNHOC124 PDX. In this model, the combination of MEK (3.5 mg/Kg) plus BEV or PTX had only marginal activity, while the combination of BEV and PTX induced tumor regression. However, tumor re-growth was observed after 47 days from the end of treatment. The addition of MEK to this doublet did not change significantly the response. Table 2 shows the T/C% values of the different experimental groups. PTX/BEV and PTX/BEV/MEK groups T/C values are 5.4% and 5.5% at day 38 respectively, corresponding to more than 90% of tumor weight inhibition. The treatment with BEV/MEK was active too (even if not superior to the previous), with a T/C% value of 27.5%, while the PTX/MEK combination was the least active (T/C% = 55.5%). All the different combinations were well tolerated with only minor changes in body weight (% body weight loss –BWL- of 1-2%, Table 2, Additional file 2: Figure S2).

**Table 2:** T/C% values and BWL% of the different treatments in the MNHOC124, MNHOC218 and MNHOC239 PDXs

	MNHOC124		MNHOC218		MNHOC239	
	T/C% (day)	BWL% (day)	T/C% (day)	BWL% (day)	T/C% (day)	BWL% (day)
PTX/BEV	5.4 (38)	-	6.6 (46)	0.8 (31)	23.8 (64)	-
PTX/MEK	55.5 (38)	-	45.2 (46)	5.6 (38)	49.2 (61)	0.3 (28)
BEV/MEK	27.5 (38)	2.3 (31)	16.2 (16)	0.2 (38)	25.5 (61)	-
PTX/BEV/MEK	5.5 (38)	1.3 (31)	0.7 (46)	4.7 (31)	13.7 (47)	-

T/C% values are considered significant of drug response when ≤42% (NCI guidelines). BWL: Body Weight Loss

**Fig. 2:**
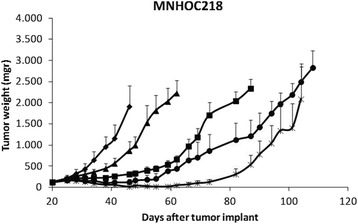
Antitumor activity of the combination of PTX, bevacizumab and MEK162 in MNHOC218 PDX model. Xenograft MNHOC218 was transplanted subcutaneously and when tumor masses reached 100-150mg, mice were randomized to receive vehicle (CTR,-♦-), or treated with Bevacizumab and MEK162 (BEV/MEK,-■-), paclitaxel and MEK162 (PTX/MEK,-▲-), paclitaxel and bevacizumab (PTX/BEV, -●-), or paclitaxel and bevacizumab and MEK162 (PTX/BEV/MEK,-x-). Data are the mean ± SE of tumor masses, as described in Materials and Methods; each group consisted of 8-10 mice

The activity of the same combinations in the MNHOC218 model is reported in Fig. 2 and Table 2, where the treatment with MEK was increased to a dose of 10mg/Kg and extended to 22 days due to the lack of toxicity observed in the MNHOC124 model. In this model the combination of PTX and MEK had marginal activity, while both BEV/PTX and BEV/MEK resulted in a significant reduction of tumor weight. The triple combination had the greater effect with a T/C value of 0.7% compared to 6.6% and 16.2% in the PTX/BEV and BEV/MEK respectively. Interestingly, the effect of the triple combination was maintained for several days. In the BEV/PTX group, the tumors started to re-grow approximately 55 days following implant, while the re-growth of the tumors was delayed of approximately 20 days in the triple combination. This increase in response was not associated with an increase in BWL%; the triple combination, in fact, induced changes in body weight comparable to the PTX/MEK combination and these changes were transient, with full recovery upon drug withdrawal (Additional file 2: Figure S2). In both these tumor models, when tumors treated with the triple combination re-grew, the growth rate was similar to the one of untreated mice, with no evidence of accelerated growth.

**Fig. 3:**
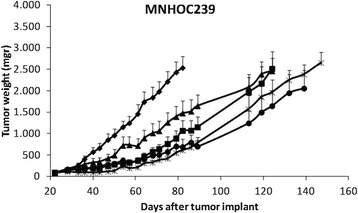
Antitumor activity of the combination of PTX, bevacizumab and MEK162 in MNHOC239 PDX model. Xenograft MNHOC239 was transplanted subcutaneously and when tumor masses reached 100-150mg, mice were randomized to receive vehicle (-♦-), or treated with Bevacizumab and MEK162 (-■-), paclitaxel and MEK162 (-▲-), paclitaxel and bevacizumab (-●-), or paclitaxel and bevacizumab and MEK162 (-x-). Data are the mean ± SE of tumor masses, as described in Materials and Methods; each group consisted of 8-10

Finally, the three drugs were tested in MNHOC239 PDX model (Fig. 3, Table 2). Overall, all the treatments displayed less activity than the one observed in the previous models. The most active combination was the triple one even if, differently from the other models, only tumor stabilization and no tumor regressions were observed. Interestingly enough, tumor stabilization was followed by a tumor re-growth (upon drug withdrawal) whose rate was lower than that of untreated controls. BEV/MEK or BEV/PTX combinations showed high activity, while only a limited one was observed in mice treated with the doublet PTX/MEK. The triple combination induced a higher regression with T/C value of 13.7%. As was for the other models, the four different combinations did not induce significant changes in animal body weights (Additional file 2: Figure S2).

We have been able to recapitulate the ovarian clinical setting in which the initial response to platinum-based therapy is generally followed by a relapse (within a variable time frame) and the re-growing masses are much less sensitive to a second challenge with platinum [14]. These preclinical PDX models represent a framework where new drugs or new drug combinations can be rapidly tested. In addition, clues of possible spectrum of toxicities could also be uncovered in this setting. We here tested the ability of the MEK inhibitor MEK162 to improve the response of second line therapy in PDX ovarian xenografts.

While in ovarian cancer paclitaxel is both used in front-line with platinum and in second line therapy, topotecan [15], pegylated liposomal doxorubicin [16] and trabectedin [17] have been approved in the platinum resistant recurrent setting with marginal activity. A recently approved targeted drug for the treatment of platinum-resistant EOC is bevacizumab, a humanized anti-VEGF monoclonal antibody with an antiangiogenic activity [3]. Indeed, four phases III trials have demonstrated a prolonged progression free survival (PFS) in patients receiving bevacizumab in combination with front-line chemotherapy (GOG protocol 18 [18] and ICON7 [19]), in combination with chemotherapy in platinum-resistant (Aurelia trial [20]) and in platinum sensitive recurrent EOC (OCEANS trial) [21]. OS did not reach a statistically significant difference, even if in subgroups analyses a trend of improved OS was found in the bevacizumab treated arm [19].

Inhibitors of MEK represent an emerging class of potentially active targeted agents for different reasons [22–24]. However, clinical activity of MEK inhibitors as single agents has been reported in malignancies with *RAS* and *B-RAF* mutations, as melanoma, while limited activity has been observed in unselected cancer patients [23].

The RAS/RAF/MEK/MAPK pathway has been shown to be activated by gene copy number aberration and or mutation in EOC [25]. It has been reported that in high grade serous ovarian carcinoma, MEK can be constitutively activated not only through BRAF but also through other MAP kinases such as MAPK8 kinase [26]. In addition, it was recently reported that high intra-tumor MAPK was an independent predictor of worse survival in EOC and that combined treatment with MEK inhibitors and fulvestran (an estrogen receptor antagonist) effectively reduced ovarian cancer xenografts growth [27]. The over activation of the pathways has been correlated with a decrease chemo-sensitivity, including to platinum containing drugs.

The hypothesis that recurrent DDP resistant ovarian xenogratfs tumors could benefit from chemotherapeutic regimens containing MEK inhibitor and drugs clinically used in a second line setting was herein tested and the results obtained clearly indicate that the combination of paclitaxel and bevacizumab is active in specific setting, confirming clinical data [18–21]. The combination of MEK162 and paclitaxel or MEK162 and bevacizumab has much less activity than the paclitaxel-bevacizumab doublet thus not supporting the use of MEK162 as doublet partner for each of this single drug. However, we have shown evidence that the triple combination in which MEK162, added on top of beva-paclitaxel combination, is indeed able to induce long lasting response in second line.

The use of PDXs to predict and test second line therapy has been recently reported in melanomas [28, 29]. In their study the PDX models were directly obtained from BRAF inhibitor-progressing tumors and gave interesting information on possible new therapies. Our study has some important differences from the one of Krepler et al. The PDXs we used were originally developed from platinum responsive patients and were used in second line after relapse from platinum therapy in vivo in nude mice. We have in this way recapitulated the clinical situation of initial response and taken the tumor soon after it relapsed. These relapsing PDXs were treated with drugs clinically used in relapsing ovarian tumors in combination with a selective MEK inhibitor. This has allowed us to confirm the good response to paclitaxel-bevacizumab and at the same time to prove that the addition of a third drug (MEK162) increased the activity with apparently no increase in toxicity.

## Conclusions

Three PDX ovarian models mimicking the clinical setting, i.e. relapsing, platinum resistant with activated AKT pathways and activated ERK, were selected to test the activity of poli-chemotherapeutic regimens containing paclitaxel, bevacizumab and a MEK inhibitor. In all the models the triple combination was well tolerated and show an antitumor activity higher than the double combinations. These results corroborate both the activity of bevacizumab in combination with chemotherapy for the treatment of ovarian tumors and that this antitumor activity can be further improved by the addition of another targeted agents (MEK inhibitor) and strongly support the use of relapsing PDXs for the discovery of new potential treatment in second line.

## Additional files

Additional file 1: Figure S1. A) Tumor growth inhibition after DDP treatment in ovarian PDXs. PDXs were treated (-○-) or not (-●-) with cDDP. The mean ± standard error of the tumor weight (mg) of each experimental group at different time points is represented. Each triangle indicates a DDP treatment (one cycle consisting of three weekly treatment), and each group consisted of 8–10 mice. B) Quantification of DDP antitumor effect in the different ovarian cancer PDXs. The histograms represent the mean ± standard error of the slope of the interpolation lines in untreated/control and DDP-treated groups ((*white box*) CTR, (*grey box*) 1st DDP cycle, and (*black box*) 2nd DDP cycle) in ovarian cancer xenografts (MNHOC124, MNHOC218, and MNHOC239) *p < 0.05, **p < 0.005, ***p < 0.0005. (PDF 386 kb)

Additional file 2: Figure S2. Body weight of animals treated with vehicle (CTR,-♦-), with Bevacizumab and MEK162 (BEV/MEK,-■-), paclitaxel and MEK162 (PTX/MEK,-▲-), paclitaxel and bevacizumab (PTX/BEV, -●-), or paclitaxel and bevacizumab and MEK162 (PTX/BEV/MEK,-x-). Data are expressed as mean±SD, each group consisted of 8-10 animals. A) MNHOC124 PDX model, B) MNHOC218 PDX model and C) MNHOC239 PDX model. (PDF 474 kb)

## Abbreviations

BEV: Bevacizumab
BWL: Body weight loss
DDP: Cisplatin
EOC: Epithelial ovarian cancer
MEK: MEK inhibitor
OS: Overall survival
PDX: Patient derived xenografts
PFS: Progression free survival
PTX: Paclitaxel
