# Supplementary figures and images for: Combination of paclitaxel, bevacizumab and MEK162 in second line treatment in platinum-relapsing patient derived ovarian cancer xenografts
Source: Mol Cancer. 2017 May 30;16:97. doi: 10.1186/s12943-017-0662-3 (PMC5450309; doi:10.1186/s12943-017-0662-3)

A)

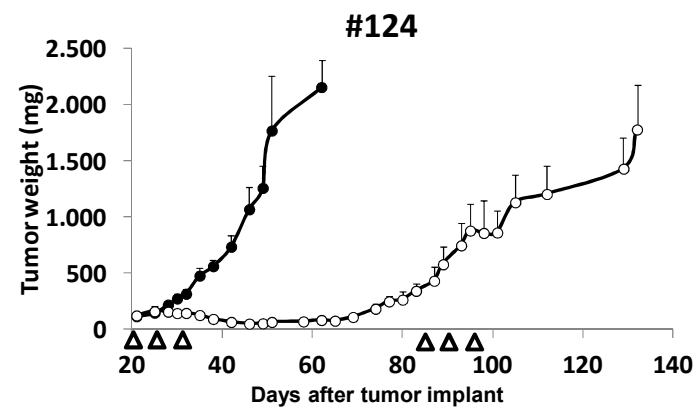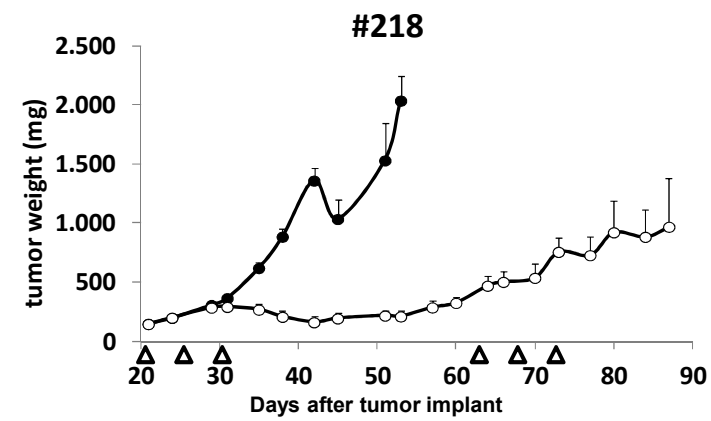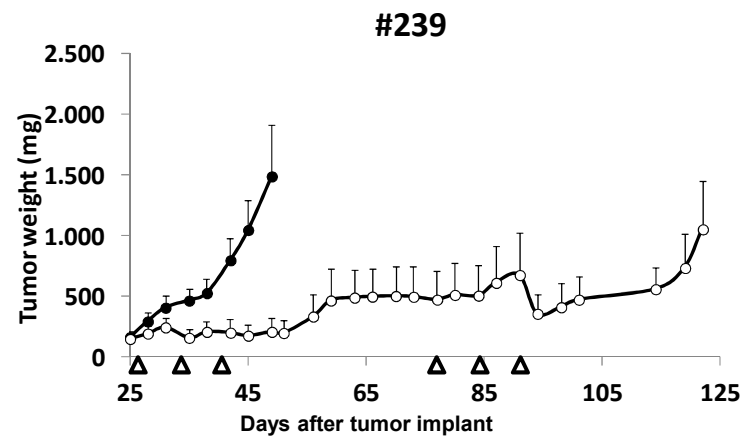

B)

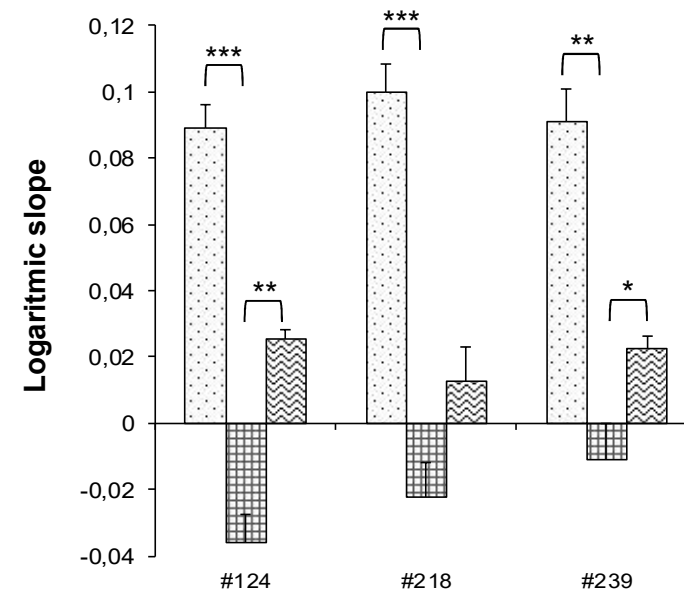

Supplement: Supplementary file 1 — A) Tumor growth inhibition after DDP treatment in ovarian PDXs. PDXs were treated (-○-) or not (-●-) with cDDP. The mean ± standard error of the tumor weight (mg) of each experimental group at different time points is represented. Each triangle indicates a DDP treatment (one cycle consisting of three weekly treatment), and each group consisted of 8–10 mice. B) Quantification of DDP antitumor effect in the different ovarian cancer PDXs. The histograms represent the mean ± standard error of the slope of the interpolation lines in untreated/control and DDP-treated groups ((white box) CTR, (grey box) 1st DDP cycle, and (black box) 2nd DDP cycle) in ovarian cancer xenografts (MNHOC124, MNHOC218, and MNHOC239) *p < 0.05, **p < 0.005, ***p < 0.0005. (PDF 386 kb) [file 12943_2017_662_MOESM1_ESM.pdf]

Supplementary figure 2

A)

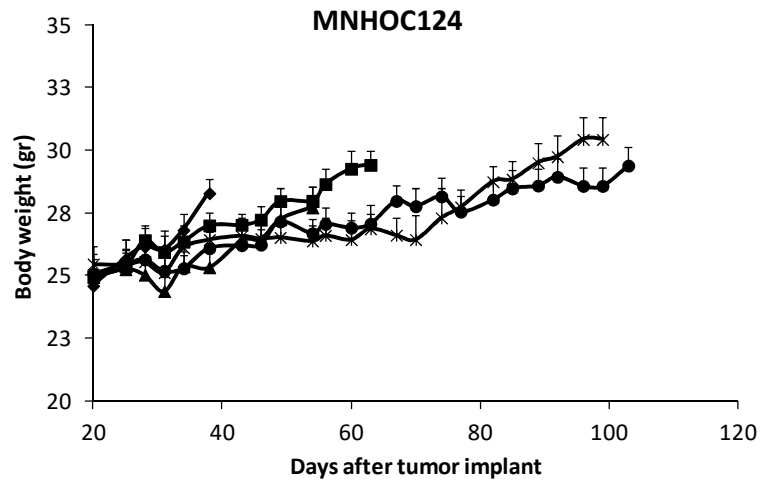

B)

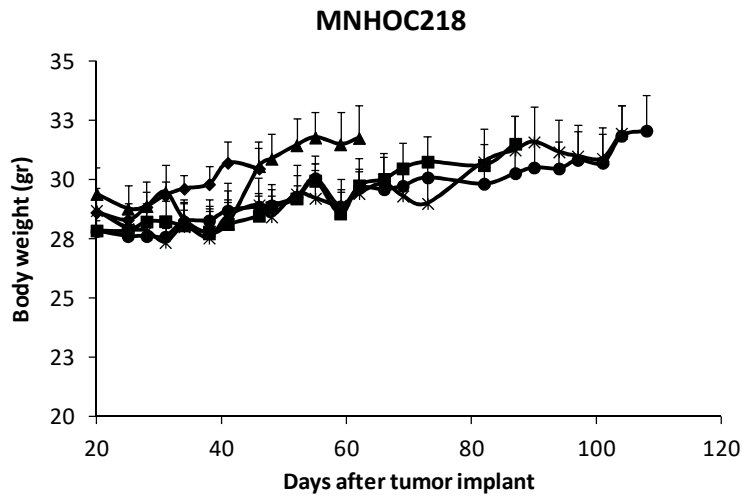

C)

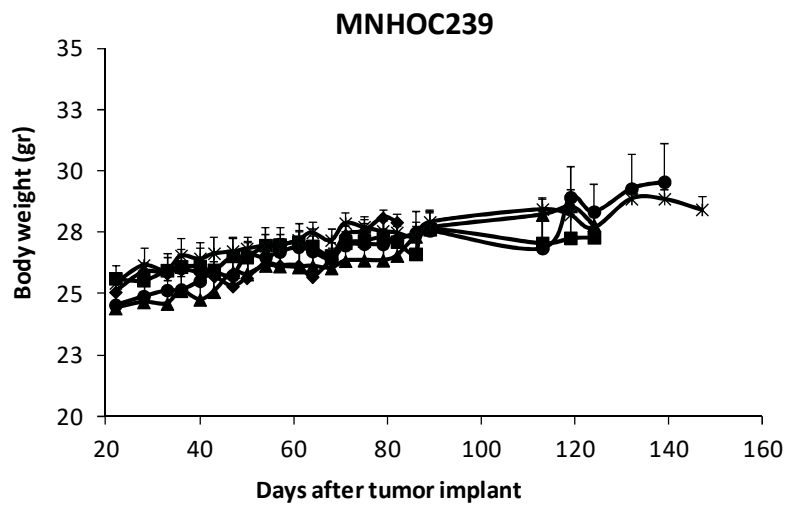

Supplement: Supplementary file 2 — Body weight of animals treated with vehicle (CTR,-♦-), with Bevacizumab and MEK162 (BEV/MEK,-■-), paclitaxel and MEK162 (PTX/MEK,-▲-), paclitaxel and bevacizumab (PTX/BEV, -●-), or paclitaxel and bevacizumab and MEK162 (PTX/BEV/MEK,-x-). Data are expressed as mean±SD, each group consisted of 8-10 animals. A) MNHOC124 PDX model, B) MNHOC218 PDX model and C) MNHOC239 PDX model. (PDF 474 kb) [file 12943_2017_662_MOESM2_ESM.pdf]
